# Large Aggregates Are the Major Soluble Aβ Species in AD Brain Fractionated with Density Gradient Ultracentrifugation

**DOI:** 10.1371/journal.pone.0032014

**Published:** 2012-02-15

**Authors:** Dag Sehlin, Hillevi Englund, Barbro Simu, Mikael Karlsson, Martin Ingelsson, Fredrik Nikolajeff, Lars Lannfelt, Frida Ekholm Pettersson

**Affiliations:** 1 Molecular Geriatrics, Department of Public Health and Caring Sciences, Uppsala University, Uppsala, Sweden; 2 Materials Science, Department of Engineering Sciences, Uppsala University, Uppsala, Sweden; Mental Health Research Institute of Victoria, Australia

## Abstract

Soluble amyloid-β (Aβ) aggregates of various sizes, ranging from dimers to large protofibrils, have been associated with neurotoxicity and synaptic dysfunction in Alzheimer's Disease (AD). To investigate the properties of biologically relevant Aβ species, brain extracts from amyloid β protein precursor (AβPP) transgenic mice and AD patients as well as synthetic Aβ preparations were separated by size under native conditions with density gradient ultracentrifugation. The fractionated samples were then analyzed with atomic force microscopy (AFM), ELISA, and MTT cell viability assay. Based on AFM appearance and immunoreactivity to our protofibril selective antibody mAb158, synthetic Aβ42 was divided in four fractions, with large aggregates in fraction 1 and the smallest species in fraction 4. Synthetic Aβ aggregates from fractions 2 and 3 proved to be most toxic in an MTT assay. In AβPP transgenic mouse brain, the most abundant soluble Aβ species were found in fraction 2 and consisted mainly of Aβ40. Also in AD brains, Aβ was mainly found in fraction 2 but primarily as Aβ42. All biologically derived Aβ from fraction 2 was immunologically discriminated from smaller species with mAb158. Thus, the predominant species of biologically derived soluble Aβ, natively separated by density gradient ultracentrifugation, were found to match the size of the neurotoxic, 80–500 kDa synthetic Aβ protofibrils and were equally detected with mAb158.

## Introduction

Soluble aggregates of the amyloid-β (Aβ) peptide have become the focus of Alzheimer's disease (AD) research as they are neurotoxic and inhibit synapse function [1], [2], [3], [4], [5], [6], [7], [8]. While CSF levels of Aβ42 declines during the presymptomatic stages of AD [9], elevated levels of soluble Aβ in the brain has been demonstrated to correlate with AD progression [10], [11], [12] and to predict synaptic degeneration [13]. In addition, an increase in soluble brain Aβ precedes plaque formation in Down syndrome brain [14]. Several different oligomeric Aβ species have been identified both *in vitro* and *in vivo* and the Aβ species responsible for neurodegeneration and synapse dysfunction has been suggested to be everything from Aβ dimers up to large protofibrils [15], [16], [17], [18], [19], [20], [21], [22], [23], [24], [25], [26], [27]. The potential importance of these Aβ species as targets for immunotherapy and biomarker assays emphasizes the need to study them in closer detail *in vivo*.

In this study the aim was to characterize the soluble pool of synthetic Aβ as well as Aβ derived from different biological samples under conditions as native as possible. Density gradient ultracentrifugation [28], unlike SDS-PAGE, size exclusion chromatography and ultra filtration, is a method where molecules are separated based on their size in a non solid matrix without any detergents or other denaturing agents. This approach is more likely to keep the Aβ aggregates intact during the analyses. ELISA quantification of different Aβ species in our centrifuged samples followed by structure analysis with atomic force microscopy (AFM) allowed us to divide the samples into four distinct fractions containing Aβ of different size and appearance. From these analyses we could conclude that large Aβ aggregates are the major Aβ species in soluble extracts from AβPP transgenic and AD patient brains.

## Results

### Characterization of fractionated synthetic Aβ

**Figure 1:**
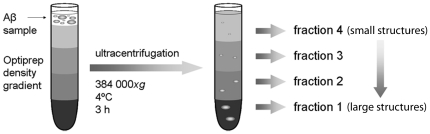
Density gradient ultracentrifugation. Schematic picture of the density gradient ultracentrifugation setup and the fractionation into four fractions, with the largest structures in the first fraction and the smallest structures in the fourth fraction.

**Figure 2:**
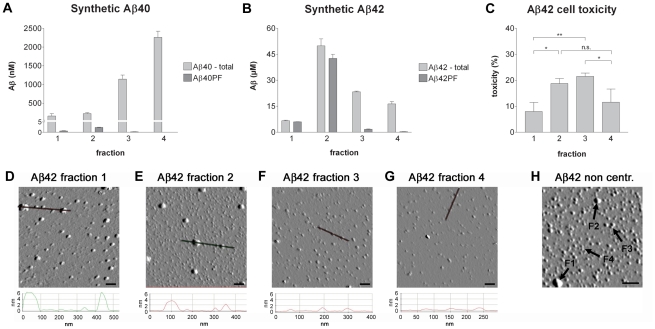
Fractionation of synthetic Aβ1–40 and Aβ1–42. Each of the four fractions of the Aβ40 and Aβ42 preparations was analyzed with ELISA for total levels of Aβ1–40 and Aβ1–42 respectively as well as for protofibril levels (A–B). Graphs show mean of three different fractionated Aβ preparations and error bars indicate the standard deviation. Equal amounts of Aβ (0.1 µM) from the Aβ1–42 fractions were also analyzed with MTT cell viability assay with PC12 cells (C). The MTT graph shows the mean of three analyses of three different fractionated Aβ1–42 preparations. The analyses are standardized with an internal Aβ control and all fractions were diluted to a final Aβ concentration of 0.1 µM and an optiprep concentration of 1.5% prior to addition to the cells. Error bars indicate the standard deviation. Aβ1–42 fractions were also analyzed with AFM (D–G). Scale bar = 100 nm. Curves under AFM images represent the topography of the area marked by a line in each image. An AFM image of a non centrifuged preparation of Aβ1–42 is shown for reference (H). Arrows point at structures resembling those present in the four different fractions of centrifuged Aβ1–42.

Two different preparations of synthetic Aβ were centrifuged in an optiprep density gradient and collected in four fractions (Fig. 1). Synthetic Aβ1–42, incubated for 30 min at 37°C, was used to obtain a wide range of soluble Aβ aggregates of different sizes in contrast to freshly dissolved synthetic Aβ1–40, which was used to represent monomeric and low-molecular weight Aβ species. These Aβ preparations were used to analyze how soluble Aβ in different aggregation states separated in the optiprep gradient. Most of the freshly dissolved Aβ1–40 was found in fractions 3 and 4, containing the smallest molecules (Fig. 2A), confirming that the Aβ1–40 preparation consists of monomers and smaller oligomers. Aβ protofibril ELISA analysis of the fractionated freshly dissolved Aβ1–40 revealed tiny amounts of Aβ aggregates in fraction 2 but nothing in fraction 3 and 4, suggesting that aggregates present in fraction 3 are too small to be detected by the conformation dependent mAb158 of the protofibril specific ELISA. As seen in figure 2B, all four fractions of Aβ1–42 contained Aβ, most of which was found in fraction 2. Most Aβ found in the two larger fractions was detected by the Aβ protofibril specific ELISA, whereas the molecules in fraction 3 and 4 also in the Aβ42 preparation were too small to be detected by the protofibril specific ELISA. Freezing of samples prior to analysis did not affect the results (data not shown).

### Different fractions' influence on cell viability

After adjusting each fraction to an optiprep density of 1.5% and an Aβ concentration of 0.1 µM, the four fractions of synthetic Aβ1–42 were analyzed with an MTT assay to test the fractions' influence on PC12 cell viability. Interestingly, the two middle fractions, containing mAb158 positive aggregates (fraction 2) and smaller mAb158 negative oligomers (fraction 3), showed a significantly higher toxicity than the other two fractions (Fig. 2C).

### Atomic force microscopy

To get an idea of the appearance of the fractionated Aβ, the four fractions of synthetic Aβ1–42 were analyzed with AFM. This analysis revealed the largest aggregates in fraction 1 with a height of 6 nm and a diameter of approximately 50 nm (Fig. 2D). The aggregates were then gradually smaller, both in height and diameter, in fraction 2 to 4 (Fig. 2E–G). Estimated molecular weights, *Mw*, of the aggregates in the different fractions were calculated with equation 1 [29] (Table 1), based on the dimensions obtained from AFM analyses.
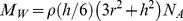
(1)In this equation, *ρ* is the protein density [30], *h* is the height and *r* is the radius of the visualized structure, measured at half the height, to compensate for tip broadening effects [29]. *N_A_* is Avogadro's constant.

**Table 1:** Molecular sizes of fractionated synthetic Aβ42, determined with AFM.

Fraction	Diameter (nm)	Height (nm)	Mw(kDa)	Aβ *n*
1	25–50	2.9–7.5	200–2000	44–440
2	25–40	1.8–3.0	80–500	18–114
3	15–25	0.8–1.2	20–80	4–18
4	12–15	0.3–0.8	5–20	1–4

The smallest structures, with an estimated size corresponding to Aβ monomers-tetramers, were to some extent visible in all fractions, possibly due to diffusion of small molecules over the gradient, caused by the high Aβ concentration in the sample. For reference, a non centrifuged sample of the Aβ1–42 preparation, aggregated for 30 min at 37°C, is displayed in Figure 2H, containing all types of Aβ species present in the fractionated samples. Examples of these structures are marked with arrows and numbers referring to the fraction in question. As a reference for size, AβPP (approx. 100 kDa) and IgG (150 kDa) both ended up in fraction 2 (data not shown).

### Fractionation of AβPP_ArcSwe_ transgenic mouse brains

**Figure 3:**
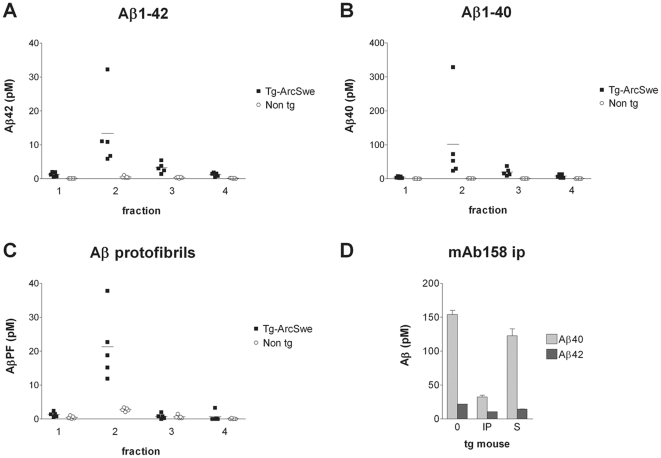
Fractionation of transgenic mouse brain extracts. Fractions of mouse brain homogenate from AβPP_ArcSwe_ transgenic mice (n = 5) and non-transgenic littermates (n = 5) were analyzed with Aβ1–42 (A), Aβ1–40 (B) and Aβ protofibril specific (C) ELISAs. Horizontal lines indicate the mean value of each group. Immunoprecipitation of pooled material from fraction 2 of transgenic mouse brain homogenate with the conformation specific Aβ protofibril selective antibody mAb158 covalently coupled to Dynabeads (0 – non immunoprecipitated sample, IP – immunoprecipitated material, S – supernatant remaining after ip) (D). Error bars indicate the standard deviation.

After verifying that the optiprep gradient is able to separate Aβ aggregates according to their size, we continued to study how a biologically derived soluble Aβ pool was separated in the same gradient. Homogenized brains from 10 month old AβPP_ArcSwe_ transgenic mice, at this age displaying robust Aβ plaque pathology [31], and non-transgenic mice were centrifuged and fractionated in the same way as synthetic Aβ. Aβ1–42 and Aβ1–40 ELISA analyses revealed that the soluble Aβ pool of transgenic mouse brain was primarily found to consist of larger aggregates ending up in fraction 2 and to some extent in fraction 3 with an Aβ42:40 ratio of approximately 1∶8 (Fig. 3A–B). No Aβ was detected in the non-transgenic mice (Fig. 3). The Aβ in fraction 2 of the mouse brain homogenate was readily detectable with the Aβ protofibril specific ELISA, whereas no Aβ protofibrils were detected in fraction 3 (Fig. 3C). To further analyze the composition of the large Aβ aggregates, immunoprecipitation was performed with the conformation dependent Aβ protofibril selective antibody mAb158 [20], covalently bound to superparamagnetic Dynabeads. The immunoprecipitate was then analyzed with highly sensitive Aβ1–40 and Aβ1–42 ELISAs. As seen in figure 3D, immunoprecipitated material from a pool of fraction 2 from five mouse brain homogenates primarily contained Aβ40, though with a lower Aβ40∶Aβ42 ratio than the starting material. Still, this result confirms that aggregates in fraction 2 of transgenic mouse brain were mainly composed of Aβ40. No Aβ was immunoprecipitated from fraction 3 (data not shown), which is in agreement with the Aβ protofibril ELISA analysis and implies that small oligomers have a different conformation than the larger protofibrillar Aβ species and that mAb158 only detects the larger aggregates.

### Fractionation of human brains

**Figure 4:**
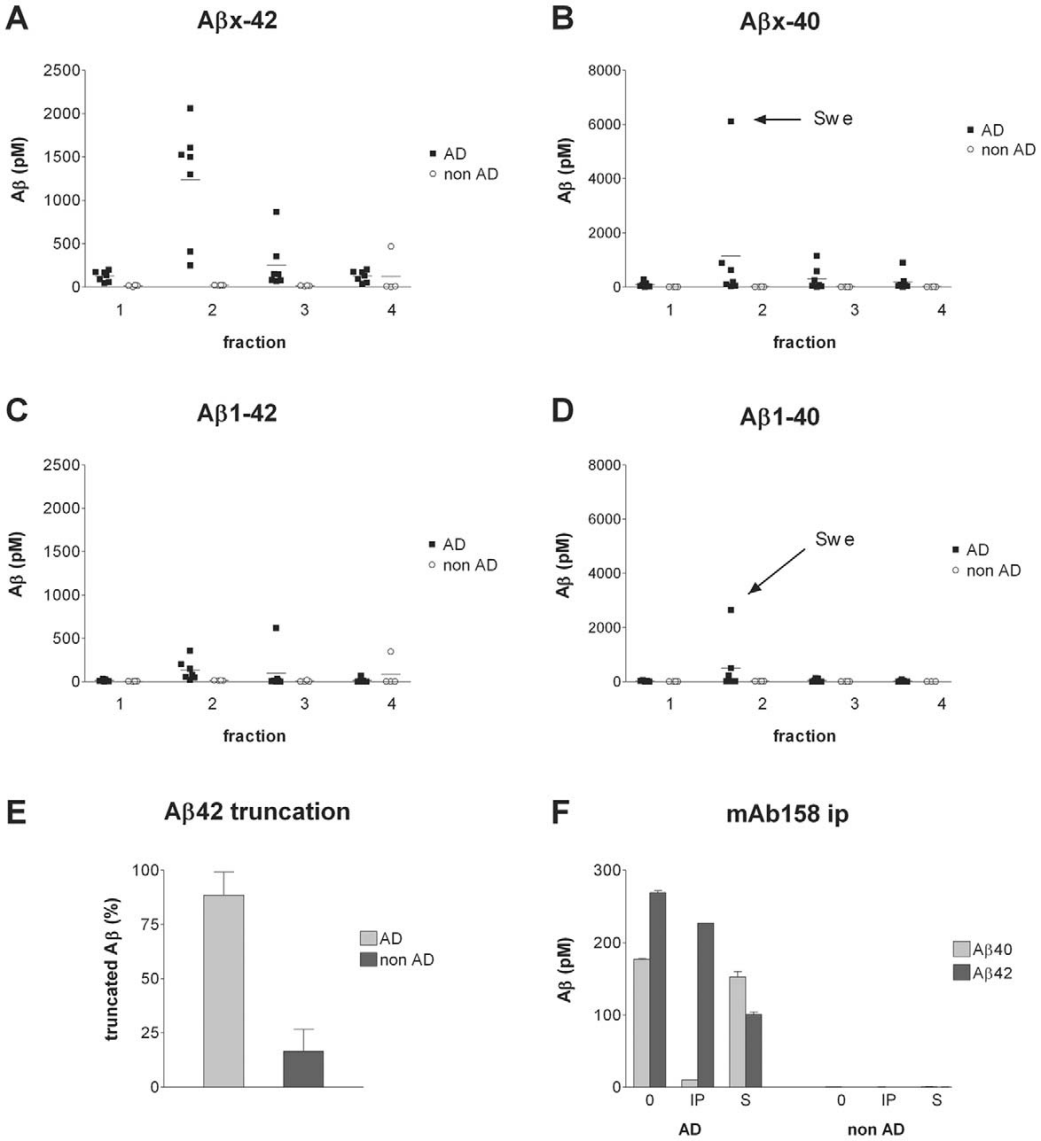
Fractionation of human brain extracts. Fractions of human brain homogenate from temporal cortex of diseased AD patients (n = 7, including one AβPP_Swe_ and one AβPP_Arc_ mutation carrier) and non-AD subjects (n = 4, one control subject and three FTD patients) were analyzed with Aβx-42 (A), Aβx-40 (B), Aβ1–42 (C) and Aβ1–40 (D) ELISAs. Results from the individual carrying the Swedish mutation is marked ‘Swe’ in the Aβx-40 and Aβ1–40 graphs. Horizontal lines indicate the mean value of each group. The level of N-terminal truncation of Aβ42 was determined as a ratio between Aβ1–42 and Aβx-42 (1-[Aβ1–42]/[Aβx-42]) (E), with error bars indicating the standard deviation. Immunoprecipitation of pooled material from fraction 2 of AD brain and non-AD brain with the conformation specific Aβ protofibril selective antibody mAb158 covalently coupled to Dynabeads (0 – non immonuprecipitated sample, IP – immunoprecipitated material, S – supernatant remaining after ip) (F). Error bars indicate the standard deviation.

To study the soluble Aβ pool in human brain, post-mortem temporal cortex tissue was taken from eleven individuals. Seven of these were post mortem confirmed AD, two of which were carriers of the Swedish or the Arctic mutation, respectively. In addition, three frontotemporal dementia (FTD) cases and one neurologically healthy control were included (table 2). Patients were diagnosed according to the NINCDS-ADRDA criteria [32]. Samples were homogenized, separated on the density gradient and fractioned as above. For the analyses, the FTD brains were included in the control group, since they were free of Aβ pathology. Accordingly, the levels of soluble Aβ40 and Aβ42 in non-AD brains were close to zero (Fig. 4A–D). All AD cases had high levels of Aβx-42, especially in fraction 2, indicating a high content of large Aβ aggregates (Fig. 4A). Also Aβx-40 levels were elevated in fraction 2 of some AD cases, especially in the brain homogenate from the Swedish mutation carrier, but there was a large variability (Fig. 4B). In line with earlier observations [33], detection with the N-terminal 82E1 antibody revealed substantially lower levels of the full length Aβ1–42 and Aβ1–40 (Fig. 4C–D), caused by a substantial degree of N-terminal truncation of soluble Aβ in AD brain, with an average of 90% truncated Aβ42 in AD brain and around 15% in non-AD brain (Fig. 4E).

**Table 2:** Summary of cases.

Case	Neuropath. diagnosis	Age at onset	Age at death	Disease duration
**001**	AD (Swe)	58	61	3
**004**	FTD	61	70	9
**005**	FTD	52	61	9
**006**	AD	80	92	12
**008**	FTD	62	66	4
**009**	AD (Arc)	56	64	8
**010**	AD	83	85	2
**011**	AD	n.d.	76	n.d.
**012**	ctrl	n.a.	88	n.a.
**013**	AD	71	77	6
**021**	AD	53	63	10

Thus, although high levels of Aβ42 were found in fraction 2 of the AD brains, no reliable Aβ protofibril ELISA analysis could be carried out. Since the Aβ protofibril ELISA is dependent on at least two epitopes with intact Aβ N-termini to generate a signal, this truncation lead to a considerable underestimation of protofibril levels. Instead, the Aβ aggregates in pooled material from fraction 2 of AD and non-AD brain were analyzed with mAb158 immunoprecipitation, requiring fewer intact N-terminal epitopes, followed by ultra sensitive Aβ1–40 and Aβ1–42 ELISA analysis. As displayed in figure 4F, the starting material contained around 40% Aβ1–40 and 60% Aβ1–42, whereas the immunoprecipitate contained 95% Aβ1–42. This was also reflected in the supernatant, where the Aβ42 level had dropped significantly, whereas Aβ40 remained more or less the same. When performing the same analysis on fraction 3 of AD and non-AD brain, no Aβ was immunoprecipitated (data not shown).

## Discussion

Many different oligomeric Aβ species have been described in the literature, but there is no consensus about which species actually exist and exert neurotoxic activity in the human brain. Small, naturally derived Aβ oligomers have been suggested to potently cause synaptic failure [26] and neuritic degeneration [34], possibly via aggregation into large protofibrils [35], and large Aβ aggregates in brain extracts and CSF have recently been associated with AD [27], [36]. Immunotherapy with antibodies able to neutralize the toxicity of oligomeric Aβ species have been suggested as a future therapy of AD [37], [38], [39], [40] and therefore, it is important to characterize the soluble Aβ pool of i.e. brain extracts from AD patients.

Here, density gradient ultracentrifugation was used to investigate the size distribution and structure of soluble Aβ from synthetic preparations and to compare them to different biological samples. This is a native and gentle method, which is important in order to maintain the structure of the Aβ aggregates. Based on observations of centrifuged synthetic Aβ, we could divide our samples into four distinct fractions, all containing Aβ species of different size and with different appearances in AFM. We have previously reported that Aβ protofibrils, recognized by our conformation dependent antibody mAb158, have an elongated structure when visualized by cryo-TEM [20]. Such protofibrils end up in fraction 2 when centrifuged on the same gradient as presented here (data not shown). Thus, the rounded shape of the larger aggregates seen in figure 2 was, although reported by others [41], somewhat unexpected. This could, to a certain degree, be an artifact caused by the attachment of samples to the mica surface, as this was not done in solution. Synthetic Aβ aggregates found in fraction 1 and 2 were mAb158 positive, whereas Aβ from fraction 3 and 4 were not, implying that the Aβ aggregates are conformationally different. In agreement with previous observations using the same method [28], we found that synthetic Aβ aggregates of intermediate size, found in fraction 2 and 3, exerted the highest toxicity to PC12 cells. Hence, the synthetic Aβ preparation appears to contain two pools of neurotoxic Aβ aggregates: a mAb158 positive pool of slightly larger aggregates and a mAb158 negative pool of smaller oligomers.

The different preparation of mouse and human brain tissue before density gradient centrifugation could potentially result in different relative amounts of Aβ being present in these samples. Despite that, there was a striking resemblance between the biologically derived Aβ, originating from AβPP_ArcSwe_ transgenic mouse brain and from AD brain, with respect to size distribution and structure. Most Aβ from these samples was found in fraction 2 and had an estimated size of 80–500 kDa. Aβ from fraction 2 of the biological samples was recognized by mAb158, implying a common structure that can be immunologically discriminated from smaller oligomers, as shown by others [42], [43], [44]. Since mAb158 does not discriminate between Aβ40 and Aβ42 aggregates *in vitro* [45], [46], this structure seems to be independent of which Aβ peptide is the main constituent of the aggregates. As previously reported, Aβ40 was the predominant Aβ peptide in AβPP_ArcSwe_ transgenic mouse brain [47] and this peptide was abundantly detected as mAb158 positive aggregates in fraction 2. The large amount of Aβ40 incorporated in aggregates may be a result of the massive overproduction of Aβ in general, caused by the Swedish mutation, in combination with the aggregation enhancing Arctic mutation. AD brains, on the other hand, contained mostly Aβ42 as described earlier [12] although a subset of them, notably the Swedish mutation carrier, had high levels of Aβ40, similar to the transgenic mice. Interestingly, Aβ aggregates, immunoprecipitated with mAb158 from fraction 2 of pooled AD brain material, contained almost exclusively Aβ42 despite a considerable amount of Aβ40 in the starting material. This result suggests that while Aβ42 in the AD brain is incorporated in large mAb158 positive aggregates, Aβ40 may have ended up in fraction 2 as monomers or small, mAb158 negative aggregates, possibly in complex with other large molecules.

Although synthetic and biologically derived Aβ may not be directly comparable, mainly because of differences in sample matrices, it is intriguing that most Aβ found in our biological samples ends up in fraction 2, where some of the most toxic synthetic Aβ species are found. This observation is in line with previous reports about size and function of Aβ aggregates, both synthetic [35], [41], [46] and from human brain tissue [27], [48].

In conclusion, although different in Aβ peptide composition and amount of N-terminal truncation, soluble Aβ aggregates from AβPP_ArcSwe_ transgenic mouse brain and AD brain, mainly found in fraction 2, appear to be similar in both structure and size, as indicated by their binding to the protofibril selective, conformation dependent antibody. Furthermore, biologically derived soluble Aβ aggregates resemble the neurotoxic aggregates found in fraction 2 of a synthetic Aβ preparation. These insights may be of relevance for the further development of immunotherapy directed against soluble species of aggregated Aβ.

## Materials and Methods

### Ethics statement

The use of human and mouse brain material was approved by the Regional ethical committee in Uppsala (decision numbers C223/8 and 2005–103). Written informed consent was obtained from all subjects (or their relatives) involved in the study.

### Density gradient ultracentrifugation

A density gradient, for fractionation of samples by ultracentrifugation, was obtained using optiprep (Sigma-Aldrich, Stockholm, Sweden) diluted in phosphate buffered saline (PBS). The gradient was prepared by layering 0.65 ml of 50% optiprep at the bottom of a 4.9 ml OptiSeal™ Polyallomer centrifuge tube (Beckman Coulter, Bromma, Sweden) followed by 0.65 ml of 40%, 1.95 ml of 30%, 0.65 ml of 20% and 0.65 ml of 10% to give a final discontinuous gradient of 4.55 ml. A 0.25 ml aliquot of sample was carefully loaded onto the top of the gradient before centrifugation. An NVT65.2 rotor (Beckman Coulter) was used, and synthetic as well as biological samples were centrifuged at 384 000×*g* for 3 h at +4°C. Fractions (1–1.65 ml, 2–0.9 ml, 3–0.9 ml, 4–1.35 ml), were collected from the bottom of the tube, aliquoted and stored at −20°C until analysis.

### Synthetic Aβ samples

Synthetic Aβ1–42 (American Peptide Company Inc., Sunnyvale, CA, USA), dissolved in 10 mM NaOH, diluted in 10× PBS to 443 µM (2 mg/ml), was incubated for 30 min at 37°C and centrifuged for 5 min at 17 900×*g* to remove any insoluble aggregates. It was then immediately applied to the density gradient for ultracentrifugation (The high concentration [443 µM] was required to perform the toxicity studies, where all fractions were diluted to the same final Aβ concentration.). Synthetic Aβ1–40 (American Peptide Company Inc.), dissolved in 10 mM NaOH, was diluted in 2× PBS to 50 µM immediately prior to centrifugation.

### Human and mouse brain homogenates

Saline perfused brain hemispheres, with a weight of approximately 150 mg, from AβPP_ArcSwe_ transgenic mice [31] (n = 5) and non-transgenic littermates (n = 5) were homogenized using a tissue grinder with teflon pestle (2×10 strokes on ice) in tris buffered saline (TBS) (20 mM tris, 137 mM NaCl, pH 7.6 and Complete protease inhibitor cocktail (Roche, Bromma, Sweden)) in a 1∶10 (tissue weight∶extraction volume) ratio. Homogenates were centrifuged at 100 000×*g* at 4°C for 1 h to obtain a preparation of TBS-soluble extracellular and cytosolic proteins. Supernatants were aliquoted and stored at −80°C until analysis. Human brain samples of approximately 500 mg, obtained from temporal cortex, were homogenized 1∶5 (tissue weight∶extraction volume) in TBS (as above) and clear homogenate supernatants were obtained by centrifugation at 16 000×*g* for 1 h at 4°C.

### Aβ40 and Aβ42 ELISA

96-well plates were coated with polyclonal antibodies specific for the C-terminal 40 or 42 Aβ neo-epitopes respectively [12] and blocked with 1% BSA in PBS. Samples were denatured by boiling 5 min in 0.5% sodium dodecyl sulphate (SDS) to avoid impaired Aβ quantification due to presence of aggregates [49]. After dilution 1∶5 in ELISA incubation buffer (PBS, 0.1% BSA, 0.05% Tween) samples were added to the ELISA plates and incubated for 2 h. A one hour incubation with biotinylated 4G8 (Nordic Biosite, Täby, Sweden), a monoclonal antibody binding to amino acid residue 18–22 within the Aβ sequence, or 82E1 (IBL, Hamburg, Germany), specific for the N-terminal Aβ neo-epitope (Aβ amino acid residue 1–5), generated by BACE cleavage of AβPP, followed by streptavidin-HRP (Mabtech AB, Nacka Strand, Sweden), and tetramethyl-benzidine (TMB) (ANL produkter, Älvsjö, Sweden) were used for detection and the optical density was measured at 450 nm. Alternatively, samples were treated as above but analyzed with Aβ1–40 ELISA kit *Wako II* or Aβ1–42 ELISA kit Wako, *High sensitivity* (Wako Chemicals GmbH, Neuss, Germany) according to the manufacturer's instructions. The optiprep matrix or the SDS (0.1%) did not disturb ELISA quantifications (data not shown).

### Aβ protofibril specific ELISA

The protofibril specific sandwich ELISA, based on mAb158 both as capturing and detecting antibody and thus excluding the risk of detecting Aβ monomers, is thoroughly described by Englund et al. [20]. In short, 96-well plates were coated with mAb158 and blocked with 1% BSA in PBS. Synthetic Aβ and mouse brain samples were diluted in ELISA incubation buffer (PBS, 0.1% BSA, 0.05% Tween), whereas human samples were diluted in ELISA plasma diluent (Mabtech AB) to avoid interference from heterophilic antibodies [50]. Samples were incubated for 2 h at room temperature (RT) before addition of biotinylated mAb158. Streptavidin coupled HRP (Mabtech AB) and TMB (ANL produkter) were used for detection and the optical density was measured at 450 nm.

### MTT cell toxicity assay

PC12 cells [51] (obtained from A–L Svensson, Uppsala university [52]) were plated at a density of 10 000 cells/well in a 96 well plate (cell+, Sarstedts, Sweden) and incubated for 18 h at 37°C in RPMI 1640 medium (Invitrogen, Stockholm, Sweden) supplemented with 10% dialyzed FBS (fetal bovine serum, Invitrogen). Aβ concentration in the different fractions was determined by Aβ42 ELISA after SDS-denaturation (see above). New aliquots of the synthetic Aβ42 fractions were thawed and added to the wells immediately after dilution to a final Aβ concentration of 0.1 µM and an optiprep concentration of 1.5%. A set of optiprep samples (1.5%) without Aβ was used as control and subtracted from the Aβ toxicity. PBS and 0.005% H_2_O_2_ were used as negative and positive controls respectively. Plates were incubated with samples and controls for 4 h at 37°C followed by addition of 3-(4,5-dimethylthiazol-2-yl)-2,5-diphenyltetrazolium bromide (MTT) at a final concentration of 0.5 mg/ml. Solubilization of the formazan product was achieved by addition of 100 µl/well of solubilization buffer (20% SDS in dimethyl-formamide, pH 4.8) followed by 24 h of incubation at 37°C. The formazan product was quantified by absorbance measurement at 570 nm. Three independent experiments were performed and standardized with an internal Aβ control (Aβ42, incubated 30 min at 37°C and centrifuged 5 min at 17 900×*g*).

### Atomic Force Microscopy

AFM analyses were carried out with an XE-150 large sample AFM system (Park Systems Corp., Santa Clara, CA, USA) equipped with a 150 mm×150 mm XY scanner. All measurements were performed at ambient temperature in true non-contact mode using silicon based AFM probes (ACTA, AppNano, Santa Clara, CA). Fractions of ultracentrifuged synthetic Aβ42 were diluted in PBS to a final concentration of 250 nM. A 10 µl aliquot of each sample was adsorbed to a freshly cleaved mica surface (Veeco, Cambridge, UK) over night at RT and then washed with distilled water and air dried. All analyses were performed with a scan rate of 1 Hz, with a set point between 0.93 and 1.79 µm and a Z servo gain between 1.07 and 1.83. Images were flattened and Sobel processed with XEI image analysis program (Park Systems Corp.). Sizes of the visualized structures were estimated using *Equation 1*, as explained in the results section.

### mAb158 immunoprecipitation

The monoclonal Aβ protofibril selective antibody mAb158 [20], [45] was covalently attached to superparamagnetic beads with the Dynabeads coupling kit (Invitrogen). mAb158-coupled beads (5 µl) were added to pooled material of fraction 2 from AD brain (n = 7), non-AD brain (n = 4) or AβPP_ArcSwe_ transgenic mouse brain (n = 3) in the presence of 0.05% Tween 20 and incubated 1 h on a shaker. After three washes in PBS with 0.05% Tween 20, beads as well as supernatant and the starting material were boiled 5 min in 0.5% SDS. The beads were removed with a magnet and samples were diluted 5 times in standard diluent and analyzed with Aβ1–40 ELISA kit *Wako II* or Aβ1–42 ELISA kit Wako, *High sensitivity* (Wako Chemicals GmbH) according to the manufacturer's instructions.
